# Yes, There Is Deqi Sensation in Laser Acupuncture

**DOI:** 10.1155/2013/198254

**Published:** 2013-02-20

**Authors:** Gerhard Litscher

**Affiliations:** Stronach Research Unit for Complementary and Integrative Laser Medicine, Research Unit of Biomedical Engineering in Anesthesia and Intensive Care Medicine, and TCM Research Center Graz, Medical University of Graz, Auenbruggerplatz 29, 8036 Graz, Austria

## Abstract

Deqi, a composite of unique sensations, is essential for clinical efficacy according to Traditional Chinese Medicine. It is described as a sensory perception of varying character and is mostly ascribed to metal needle acupuncture. However, it can also be elicited by different kinds of laser acupuncture stimulation. This short paper summarizes the current scientific status of deqi in laser stimulation. Different kinds of laser acupuncture are described in a comprehensive form, and the most interesting studies concerning deqi and laser acupuncture are presented.

## 1. Introduction

In relation to acupuncture research, the term “deqi” is described as a sensory perception of varying character [1]. However, deqi sensations can also be elicited without any cutaneous sensory input. Some authors conclude that deqi might be a central phenomenon of awareness and consciousness, and that its relevance should be taken into account, even in clinical studies [1].

**Figure 1:**
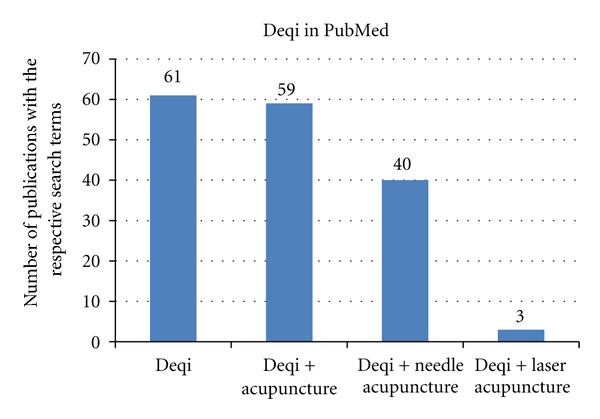
The PubMed literature search for the terms related to deqi. Note that, at the moment (Dec 3rd, 2012), there are only 3 articles mentioning deqi in connection with laser acupuncture [1, 11, 12].

The literature search in the commonly used database PubMed yielded the results demonstrated in Figure 1.

This review article summarizes the current scientific status of deqi in laser acupuncture in a comprehensive form and is aimed at motivating the readers to perform more research on this very interesting topic for acupuncture.

## 2. Different Kinds of Laser Acupuncture

Since ancient times, metal needles are the commonly used device for stimulating acupuncture points and eliciting a needle-specific deqi sensation. However, there are also new optical stimulation methods which were scientifically investigated for the first time by our research group within the last years [2]. These methods will be described in the following.

### 2.1. Violet Laser Acupuncture

Up to now, violet lasers are used only in special areas in medicine [3, 4], because it is a new and still expensive invention. In acupuncture research, violet laser was applied only in a few scientific investigations until now, which were published by our research group [5–10].

Violet laser needle acupuncture is a new optical method for stimulating different acupuncture points continuously and simultaneously. A wavelength of 405 nm, an output power of 110 mW, and a diameter of 500 *μ*m were used for our experimental investigations. The system consists of 10 semiconductor injection laser diodes.

Each single needle can emit a different wavelength. We used a continuous wave mode (CW). Due to coupling losses, the output at the tip of the needle is about 100 mW. Irradiation usually lasts 10 min (600 sec), and therefore, optical power density was very high (range: kJ/cm²) [9]. The violet laser needles are placed vertically at the skin and trigger painless but perceptible stimulation at the acupuncture point.

Violet laser acupuncture was made possible only due to latest inventions. Nakamura et al. [13] developed small, convenient blue and violet lasers which have not been available before. The acupuncture laser equipment used in our studies operates, as already mentioned before, at a wavelength of 405 nanometers. It is worth noticing that this wavelength is not in fact blue, but appears to the eye as violet, a color for which the human eye has a very limited sensitivity.

The violet laser does not have similar penetration depth in human skin as, for example, the red or infrared laser described in the next subsection (violet: approximately 2 mm versus red/infrared: 2-3 cm [5, 14, 15]); however, there is an evoked deqi sensation, which is a prerequisite for effective acupuncture stimulation [2].

### 2.2. Red and Infrared Laser Acupuncture

“The first bichromatic laser needles (685 nm and 785 nm) were developed at the University of Paderborn, Germany (Dr. Detlef Schikora), and the first clinical investigations were performed in Lauenförde, Germany (Dr. Michael Weber). The first scientific experiments and publications on this field of research started in 2002 at our Medical University in Graz, Austria [14, 16–20].

**Figure 2:**
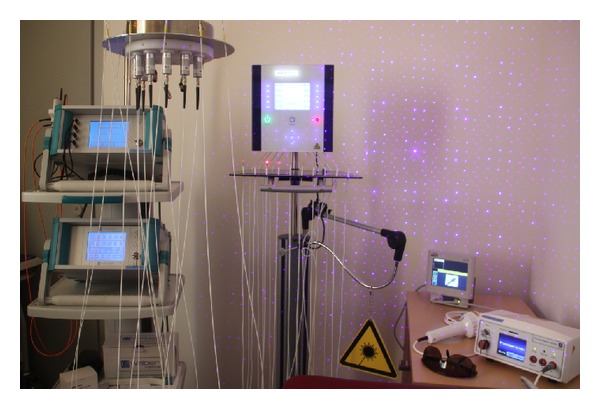
Different systems for laser acupuncture at the Medical University of Graz.

Multichannel laser needle acupuncture allows the simultaneous stimulation of individual point combinations (Figure 2) [14, 17]. Variations and combinations of acupuncture points according to TCM are possible on the body or at the ear and hand using Korean or Chinese hand acupuncture. The bichromatic laser needle method is based on systems with 8–12 separate semiconductor laser diodes and emission wavelengths of 685 nm and 785 nm. The system consists of flexible optical light fibers, which conduct the laser light with minimal loss to the laser needle. Thus, a high optical density can be achieved at the distal end of the laser needle. The intensity of the laser needles is optimized in such a way, so that the volunteer or patient does not immediately feel the activation of the needle (30–40 mW per needle; diameter 500 *μ*m; duration 10 min; power density ~20 J/cm^2^ per acupuncture point). More details regarding this method are described in previous studies and books [14, 19, 20] [2].

To the best of our knowledge, there are no studies concerning deqi and green laser or yellow laser. Green laser has a very low penetration depth and is suitable mainly for ear acupuncture or other superficial points. Yellow laser has not been used in acupuncture research up to now.

## 3. Deqi Sensation in Laser Acupuncture

Deqi is described by patients and volunteers as heaviness or like an electrical current running along the treated meridians. If red (685 nm) or infrared (785 nm) lasers are used, the patients normally do not notice when the laser is started. So, in the beginning of the treatment, they also do not feel any deqi sensation. Several minutes later (5–10 min), many patients report a pleasant warm and sometimes vibrating feeling in some treated areas [21].

In an experimental pilot study, we found that violet laser stimulation increases temperature (mean ~1.5°C) and microcirculation (mean ~20%) at the acupoint Hegu (LI.4) significantly and immediately (1 min) after stimulation onset [5]. The main interesting finding of our second publication concerning violet laser acupuncture was that heart rate decreases significantly within an interval of 5 min after violet laser stimulation onset at the acupoint Neiguan (PC.6) [2, 6]. Five interesting studies performed recently [7–10, 22] are also related to violet laser acupuncture.

According to Traditional Chinese Medicine, one must first obtain deqi sensation for acupuncture to be effective. In some studies, we could demonstrate that initial stimulation with a metal needle is stronger than the initial stimulation with a laser, but it fades earlier (it is like a spike). Laser needle stimulation is initially not as strong as the metal needle stimulation, but it continues to rise throughout the entire treatment—and it can also elicit deqi sensation [14, 21]. David Rindge, an oriental medical doctor and licensed acupuncturist, stated in *Acupuncture Today* [21]: “By the way, I have treated myself with laser needles, and I have felt this smooth deqi sensation, too.”

In a research article published in Evidence-based Complementary and Alternative Medicine, Beissner and Marzolff [23] recently (2012) described sketches of acupuncture sensations of healthy volunteers after laser needle acupuncture. Since deqi can be subtle, they tried to reduce the confounding impact of external stimuli by carrying out the experiment in a floatation tank under restricted environmental stimulation. More than 80% of the subjects experienced deqi after laser acupuncture, that is, they described line-like or two-dimensional sensations, although there were some minor doubts that these were related to the laser stimulation [23].

One limitation of our studies is that we did not quantify deqi sensation, for example, as a percentage of how many people felt deqi in laser versus metal needle acupuncture up to now. It is clear, however, that the percentage will be much smaller during laser acupuncture. At the moment, we also have no comparison of healthy volunteers versus patients on this topic.

Beside subjective and objective data that there is also a clear deqi sensation evoked by laser acupuncture, another related study published recently by our group is of particular interest [24]. In this study, we reported small, but reproducible human cerebral evoked potentials after bilateral, nonperceptible laser needle (658 nm, 40 mW, 500 *μ*m, 1 Hz) irradiation of the Neiguan acupoint (PC6). These findings indicate that exposure to laser needle stimulation with a frequency of 1 Hz can modulate the ascending reticular activating system and can possibly act as further explanation for deqi-like sensations in laser acupuncture stimulation. Further investigations concerning this interesting topic of research are in progress.
